# Gay App Use, Sexuality Traits, and High-Risk Sexual Behaviors Among Men Who Have Sex With Men in China: Mediation Analysis

**DOI:** 10.2196/49137

**Published:** 2023-11-01

**Authors:** Rui Luo, Zhi Xie, Vincent M B Silenzio, Yun Kuang, Dan Luo

**Affiliations:** 1 Department of Medical Statistics School of Public Health Sun Yat-Sen University Guangzhou China; 2 Department of Social Medicine and Health Management Xiangya School of Public Health Central South University Changsha China; 3 Changsha Center for Disease Control and Prevention Changsha China; 4 Department of Urban-Global Public Health Rutgers School of Public Health Rutgers University Newark, NJ United States; 5 Changsha Zonda-sunshine Social Work Center Changsha China

**Keywords:** geosocial networking apps, men who have sex with men, respondent-driven sampling, high-risk sexual behaviors, sexuality traits, mobile phone

## Abstract

**Background:**

Gay geosocial networking apps, also known as “gay apps,” have gained increasing popularity in the men who have sex with men (MSM) community. Certain sexuality traits and gay app use are both associated with high-risk sexual behaviors among MSM. However, little is known about the underlying mechanism of such relationships.

**Objective:**

Based on the uses and gratifications theory, this study aimed to test the mediation effect of gay app use on the relationship between sexuality traits (sexual compulsivity and sexual sensation seeking) and high-risk sexual behaviors (multiple sexual partners and unprotected anal intercourse) among MSM.

**Methods:**

A cross-sectional, multicenter study was conducted in Wuhan and Changsha, China, from August to October 2020. A representative sample of 402 MSM was recruited through respondent-driven sampling. A self-administered web-based structured questionnaire was used to collect data on sociodemographic information, high-risk sexual behaviors, gay app use, sexual compulsivity, and sexual sensation seeking. Path analysis was conducted to assess the mediation effect.

**Results:**

Our study revealed that 67.42% (n=271) of MSM used gay apps for seeking potential sexual partners, with 37.06% (n=149) of them engaging in unprotected anal intercourse, and 45.42% (n=218) of them having multiple sexual partners. Of the participants, 17.16% (n=69) reported significant sexual compulsivity, while 29.10% (n=117) reported significant sexual sensation seeking. Notably, gay app usage partially mediated the relationship between sexual compulsivity and multiple sexual partners but fully mediated the relationship between sexual compulsivity and unprotected anal intercourse. Furthermore, gay app usage partially mediated the relationship between sexual sensation seeking and multiple sexual partners but fully mediated the relationship between sexual sensation seeking and unprotected anal intercourse.

**Conclusions:**

High-risk sexual behaviors are common among MSM. Most MSM rely on gay apps to find sexual partners, which, when combined with higher levels of sexual compulsivity and sexual sensation seeking, can increase the likelihood of engaging in high-risk sexual behaviors. Therefore, interventions aimed at reducing these behaviors among MSM should focus on addressing the use of gay apps, while also considering the influence of their sexuality traits on gay app use.

## Introduction

In recent decades, while the overall HIV/AIDS epidemic in China has remained low, the rate of infection has risen significantly in specific populations. By the end of 2020, there were an estimated 1.053 million people living with HIV in China [1]. Men who have sex with men (MSM) are considered a high-risk group for HIV infection due to their high-risk sexual behavior, primarily anal intercourse, and certain subcultural characteristics that may contribute to increased risk [2]. A recent review of 355 studies covering 59 cities from 30 provinces and municipalities of China reported that the overall national prevalence of HIV among MSM from 2001 to 2018 was 5.7% (95% CI 5.4%-6.1%), with an increasing trend over time [3]. HIV testing is one of the most cost-effective strategies for HIV prevention and control in China and has been shown to reduce health care costs [4]. Frequent HIV testing enables early detection of infection and allows for timely access to medical and social services, which not only improves the prognosis of the infected person but also helps prevent further spread of HIV [5].

Due to the rapid development of internet technology and shifts in social culture, the dating habits of MSM in China have undergone significant changes, which has contributed to the spread of sexually transmitted infections (STIs), including HIV, among this group. In the 1980s, bathrooms, where homosexual individuals congregated, were the primary location for MSM to find sexual partners, and these became one of the main sources of STI transmission [6]. Since the late 1990s, gay dating websites have been widely used in China, and nearly half of Chinese MSM have used these websites to find partners and engage in sexual activities [7]. With the advent of GPS smartphones and the growth of various smartphone apps, gay geosocial networking smartphone apps (gay apps) have gained widespread popularity in China and have become the main tool used by MSM to find and connect with sexual partners [8]. For instance, the gay app “Blued,” designed specifically for MSM, was launched in 2012 and has since become the largest gay social networking platform in China. With 40 million registered users worldwide and 8 million monthly active users, Blued has been recognized as one of the largest gay apps in the world [9].

Compared to traditional venues and websites, gay apps offer a more convenient and faster way for MSM to find potential sexual partners. With the GPS positioning function, users can easily find partners in their geographical area. Mobile phones’ portability means users can check for nearby partners at any time and place, and easily engage in offline interactions. Additionally, gay apps are often free, making them more convenient and accessible than traditional dating websites that usually require a subscription fee. All these characteristics have made gay apps popular in the MSM community, which has brought about significant challenges in HIV prevention. A review has identified a variety of high-risk sexual behaviors associated with gay app use among MSM, including the increased number of sexual partners, unprotected anal intercourse, and drug use [10]. Previous studies have shown that unprotected anal intercourse among MSM has increased from 17% to 67% after using gay apps [11,12] and that gay app users have more sexual partners than nonusers [13].

Studies have shown that MSM with certain sexuality traits, including sexual compulsivity and sexual sensation seeking, are more likely to engage in high-risk sexual behaviors. Sexual compulsivity is a persistent, repetitive, and intrusive urge to engage in specific acts in a ritualized or routine manner [14]. It is characterized by intrusive sexual fantasies that increase in frequency and intensity, causing pain or problems at personal, social, and professional levels [15,16]. Sexual compulsivity may put individuals at risk of engaging in high-risk sexual behaviors and contracting HIV. Sexual sensation seeking is characterized by the desire for diverse, novel, and complex sexual experiences and the willingness to take physical and social risks to attain them [17]. A previous study found that MSM with strong sexual sensation seeking were less concerned about the consequences of their sexual behaviors, such as engaging in unprotected anal intercourse to fulfill their desire for sexual stimulation [18]. Both sexual compulsivity and sexual sensation seeking are more prevalent among MSM compared to the general population [19] and are crucial for HIV prevention and control [20]. However, little attention has been paid to these sexuality traits of MSM and the potential health risks behind them.

The uses and gratifications theory can be applied to examine the associations between sexual traits, gay app use, and high-risk sexual behaviors among MSM. Unlike the traditional mechanic approach viewing individuals as passive media consumers, the uses and gratifications theory views media consumers as goal-directed, purposive, and motivated individuals with specific needs and considers their media usage as a means to fulfill these needs [21]. According to the uses and gratifications theory, media use is determined by several key factors including “people’s needs and motives to communicate, the psychological and social environment, the mass media, functional alternatives to media use, communication behavior, and the consequences of such behavior” [22]. Based on the uses and gratifications theory, MSM with high levels of sexual compulsivity and sexual sensation seeking may turn to gay apps to satisfy their sexual needs, potentially raising their chances of engaging in high-risk sexual behaviors. To validate this theory, we conducted this study to examine the relationship between sexuality traits (sexual compulsivity and sexual sensation seeking), gay app use, and high-risk sexual behaviors (such as unprotected anal intercourse and having multiple sexual partners) among MSM. The goal is to provide a fresh perspective and guidance for effective and practical HIV prevention and control in this population.

## Methods

### Study Settings and Participants

Both Wuhan and Changsha are provincial capital cities located in Central China, with an estimated web-based MSM population of around 20,000, which is among the highest in China [23]. In these 2 cities, there are respectively 2 and 3 social organizations dedicated to providing local MSM with rapid HIV testing, counseling, and sexual health education. This study selected 2 of these organizations with which we have established long-term collaborations as study sites. These 2 social organizations are located in Wuhan and Changsha, specializing in providing health-related services to the MSM community, and closely collaborating with the local Center for Disease Control and Prevention (CDC) to offer HIV-related counseling and rapid HIV testing services. Visitors who test positive during the initial rapid screening are referred to the local CDC’s HIV Voluntary Counseling & Testing clinic for confirmatory testing. We collaborate with these 2 social organizations to recruit study participants, as they have a strong reputation and are highly regarded within the local MSM community.

Participants were consecutively recruited from August 1 to October 30, 2020. To be eligible, participants had to meet the following criteria: (1) aged ≥18 years, (2) male, and (3) having engaged in anal intercourse with a man in the past 6 months. Participants who were cognitively impaired or unable to understand the questionnaire were excluded. The social organizations’ staff approached eligible seeds during their HIV counseling visits and explained to them our study purpose and procedure in detail. Those who were interested in participating in the study were then referred to our research team. After providing informed consent, the eligible participants were invited to complete a battery of web-based questionnaires with our research team.

### Study Design and Process

**Figure 1:**
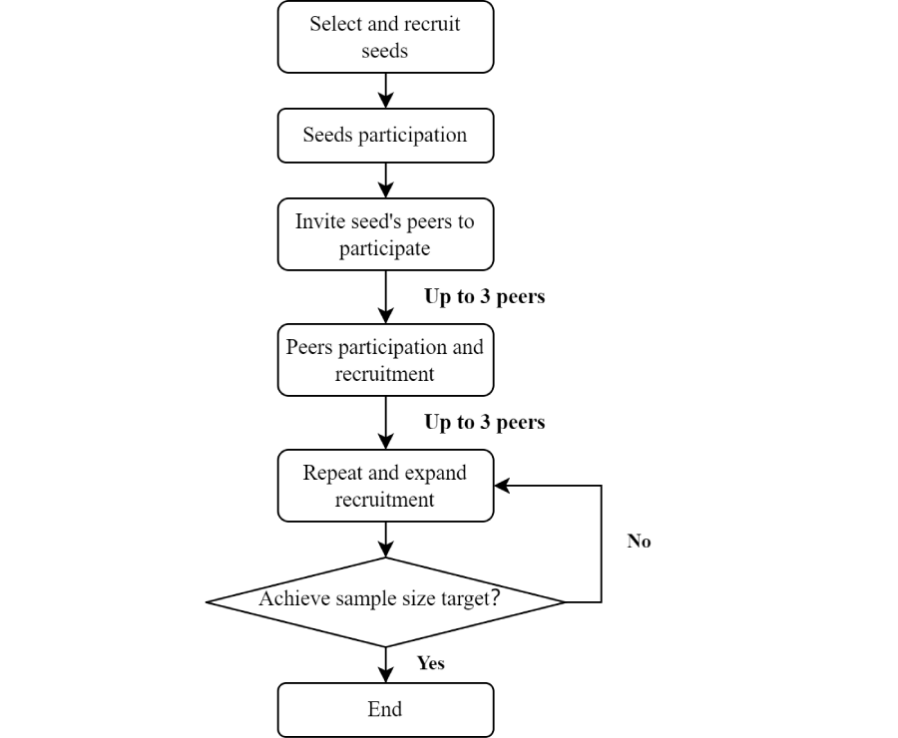
Respondent-driven sampling flowchart of the cross-sectional study among men who have sex with men in China from August 1 to October 30, 2020.

In this study, respondent-driven sampling (RDS) was used to ensure that the participants were representative. RDS has been widely used in recruiting hidden populations including MSM [24-26] and reduces biases that are common in other nonrandom sampling methods, such as snowball sampling [27,28]. The study began by recruiting 8 participants (seeds) with different occupations, such as gay bar owner, student, etc, and larger social networks among MSM. The basic characteristics of the 8 seeds are shown in Table 1. To ensure diversity among participants, the seeds did not know each other, and their social networks did not overlap. After completing the questionnaire, the seeds were given 3 coupons and asked to invite a maximum of 3 eligible peers from their gay social network to participate. The flowchart of participant recruitment is shown in Figure 1. Participants would add the study’s official WeChat account using the information on the coupon. After confirming their identity, the investigator would instruct them to complete the web-based questionnaire. After completing the questionnaire and passing quality control, each participant received 20 ¥ (approximately converted to US $2.7) as compensation. The process would then be repeated, with participants receiving 3 coupons to invite 3 more peers until the desired sample size was reached.

**Table 1:** Basic characteristics and social network size of seeds.

Seed ID	City	Age (years)	Social network size	Occupation
A	Changsha	26	182	Accountant
B	Changsha	34	300	Bar owner
C	Changsha	31	127	Lawyer
D	Changsha	23	80	College student
E	Wuhan	27	76	High school teacher
F	Wuhan	26	300	Bar owner
G	Wuhan	22	300	Nongovernmental organizations (NGO) member
H	Wuhan	20	144	College student

### Measurements

#### Sociodemographic Characteristics

Sociodemographic information was collected by a questionnaire, which included age (18-22, 23-27, and ≥28), marital status (married and single), sexual orientation (heterosexual, bisexual, and homosexual), monthly income (≤3000 ¥ and ＞3000 ¥ [US $411]), education (college or higher and high school or lower), employment (employed, unemployed, and students), and living status (living with others and living alone).

#### Gay App Use

Participants’ gay app use behavior in the past 6 months was assessed by 1 question, “How often have you used gay apps to find sexual partners in the past six months?” Optional frequencies included “never,” “1 day/several months,” “1 day/month,” “2-4 days/month,” “1-3 days/week,” “4-6 days/week,” and “every day.” Among these, we listed some commonly used gay apps in China, such as Blued, Aloha, and Jack’d, to aid participants’ recall.

#### High-Risk Sexual Behaviors

Participants’ high-risk sexual behaviors over the past 6 months were collected by 2 questions. The first question asked about participants’ usage of condoms during anal intercourse, “In the past six months, how often have you typically used condoms during all anal intercourse?” with optional frequencies ranging from “never” to “always.” The second question asked about the number of male sexual partners, “In the past six months, how many men have you had anal intercourse with?” Participants were allowed to provide a specific number equal to or greater than 1.

#### Sexual Compulsivity

Sexual compulsivity was assessed using the Sexual Compulsivity Scale (SCS) [29]. The scale is a 4-point Likert scale with 10 items, ranging from 1 (strongly disagree) to 4 (strongly agree) [29]. The total score ranges from 10 to 40, with a higher score indicating a higher degree of sexual compulsivity. A score of more than 24 was defined as having significant sexual compulsivity [30]. The scale has been used in Chinese MSM and has good reliability and validity [31]. In this study, the SCS showed good internal consistency with a Cronbach α of .92.

#### Sexual Sensation Seeking

Sexual sensation seeking was assessed using the sexual sensation seeking scale (SSSS) [17]. The scale has 11 items that measure a person’s tendency to seek sexual excitement and engage in novel sexual experiences on a 4-point Likert scale ranging from 1 (not at all like me) to 4 (very like me) [17]. The total score ranges from 11 to 44, with a higher score indicating a higher degree of sexual sensation seeking. Significant sexual sensation seeking was defined as those with a total score greater than 29, which is the 75th percentile of the scale in this study. This scale has been used in Chinese MSM and has good reliability and validity [32]. In this study, the SSSS showed good internal consistency with a Cronbach α of .90.

### Statistical Analysis

Descriptive statistics, such as frequencies and percentages, were calculated for categorical variables, and means and SDs were calculated for continuous variables. The RDS method allowed us to control for selection bias and make statements about the population without being restricted to the sample [33]. To account for the size of participants’ social networks, we adjusted the analysis and calculated the corresponding 95% CIs. All analyses were performed by the RDS package in R (version 4.1.3; R Core Team).

The path analysis was conducted to assess the relationship between psychosexual characteristics (sexual compulsivity and sexual sensation seeking), gay app use, and high-risk sexual behavior (unprotected anal intercourse and multiple sexual partners). The mediating effect of gay app use on the relationship between psychosexual characteristics and high-risk sexual behavior was tested using the nonparametric percentile bootstrapping method. The statistical significance of the estimates was evaluated based on a probability criterion of *P*<.05. The data analysis was performed using Amos (version 23.0; IBM Corp).

### Ethical Considerations

The study was approved by the Ethics Review Committee of Xiangya Public Health, Central South University (XYGW-2020-49). By signing the electronic informed consent form, participants acknowledged that their participation was voluntary and that they could withdraw at any time without any negative consequences. All personal information collected from the participants were kept confidential and stored securely in encrypted storage spaces. The results of the study were presented in a way that ensured the participants’ anonymity and protected their privacy.

## Results

### Recruitment Situation

**Figure 2:**
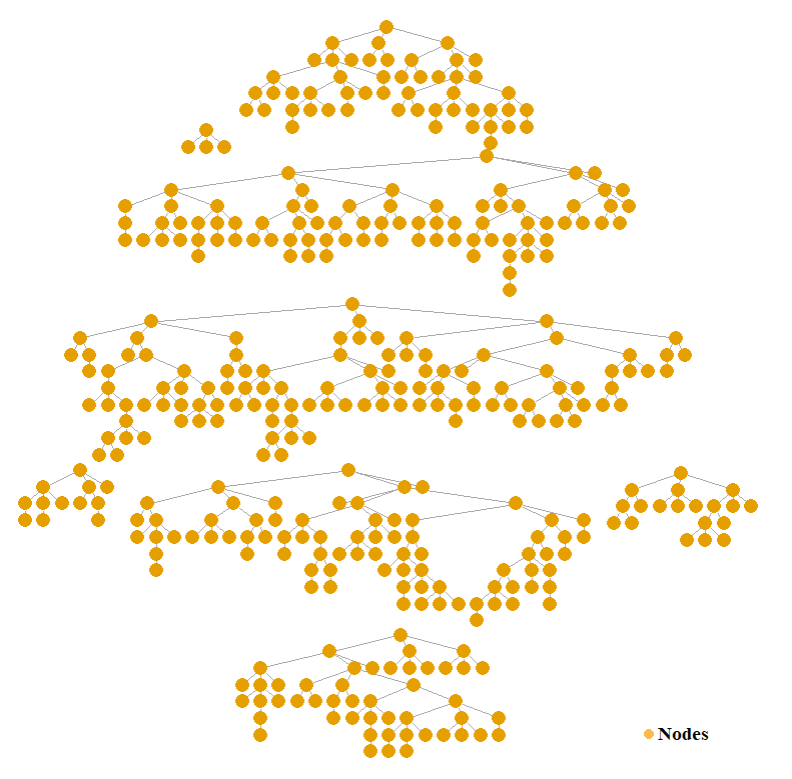
Tree diagram of respondent-driven sampling recruitment results of the cross-sectional study among men who have sex with men in China from August 1 to October 30, 2020.

Figure 2 shows the tree diagram of RDS recruitment results. The 8 seeds recruited a total of 402 MSM through RDS, with the longest recruitment wave consisting of 10 waves. The maximum number of individuals recruited by a single seed was 102. The study showed that as recruitment progressed, the basic characteristics of MSM, such as sexual orientation, unprotected anal intercourse, and multiple sexual partners, reached a balance at the third, fifth, and fourth layers, respectively (with a mean absolute difference of less than 2%). Additionally, the median size of social networks in this survey was 24 (IQR 10-60)individuals. The adjusted composition ratio revealed that 90.9% (n=199) of MSM had social networks with 24 or fewer individuals, as shown in Multimedia Appendix 1. Furthermore, we have provided a regional map of the sample’s household registration locations. Please refer to Multimedia Appendix 2 for details.

### Participant Characteristics

Table 2 shows the sample characteristics with and without adjustment. Half of the participants (53.73%, n=216) were under 23 years old, with 98.26% (n=395) of them being single and only 7 participants being married. Most of the participants (89.05%, n=358) had a bachelor’s degree or higher and 47.52% (n=191) had a monthly income of more than 3000 ¥ (~US $411). Most participants (81.09%, n=326) self-identified as homosexual. In terms of gay app use, most participants (67.42%, n=271) have used gay apps for the past 6 months, including 35 (8.71%) who used it everyday. A total of 69 (17.16%) participants reported significant sexual compulsivity and 113 (28.11%) reported significant sexual sensation seeking. Regarding high-risk sexual behaviors, 149 (37.06%) participants reported engaging in unprotected anal intercourse and 218 (54.23%) reported having multiple sexual partners in the past 6 months.

**Table 2:** Descriptive analysis of sociodemographic information and major variables of the cross-sectional study among men who have sex with men in China from August 1 to October 30, 2020.

Variables			RDS^a^ crude, n (%) (N=402), n (%)		RDS adjusted^b^, % (95% CI)
**Age (years)**					
	18-22	216 (53.7)		50.0 (43.7-56.2)	
	23-27	134 (33.3)		31.4 (25.5-37.3)	
	≥28	52 (12.9)		18.6 (14.6-22.7)	
**Marital status**					
	Married	7 (1.7)		3.3 (2.6-4.1)	
	Single	395 (98.3)		96.7 (95.9-97.4)	
**Education**					
	College or higher	358 (89.1)		84.4 (81.2-87.6)	
	Senior or lower	44 (11.0)		15.6 (12.4-18.8)	
**Employment status**					
	Employed	222 (55.2)		52.2 (45.9-58.5)	
	Unemployed	15 (3.7)		2.8 (1.0-4.6)	
	Students	165 (41.1)		45.1 (38.8-51.4)	
**Monthly income**					
	≤¥ 3000 (US $411)	211 (52.5)		59.2 (53.0-65.4)	
	＞¥ 3000 (US $411)	191 (47.5)		40.8 (34.6-47.0)	
**Sexual orientation**					
	Heterosexual	3 (0.8)		3.3 (2.9-3.7)	
	Bisexual	733 (18.2)		21.7 (16.8-26.6)	
	Homosexual	326 (81.1)		75.0 (70.1-79.9)	
**Living status**					
	Living with others	292 (72.6)		76.8 (71.6-82.0)	
	Living alone	110 (27.4)		23.2 (18.0-28.4)	
**Gay app use**					
	Never	131 (32.6)		45.8 (39.6-51.9)	
	1 day per several months	55 (13.7)		12.5 (7.8-17.2)	
	1 day per month	37 (9.2)		6.1 (2.5-9.7)	
	2-4 days per month	67 (16.7)		14.2 (9.7-18.7)	
	1-3 days per week	51 (12.7)		8.2 (4.4-12.0)	
	4-6 days per week	26 (6.5)		4.5 (1.9-7.2)	
	Everyday	35 (8.7)		8.6 (5.5-11.8)	
**Sexual compulsivity**					
	Significant	69 (17.2)		12.9 (8.0-17.8)	
	No significant	333 (82.8)		87.1 (80.3-17.8)	
**Sexual sensation seeking**					
	Significant	117 (29.1)		21.2 (15.7-26.8)	
	No significant	285 (70.9)		78.8 (73.2-84.3)	
**Unprotected anal intercourse**					
	Always	23 (5.7)		15.2 (12.4-18.1)	
	Often	14 (3.5)		4.3 (2.0-6.7)	
	Half of time	37 (9.2)		5.5 (1.9-9.2)	
	Sometimes	75 (18.7)		19.4 (15.0-23.9)	
	Never	253 (62.9)		55.4 (49.5-61.3)	
**Multiple sex partners**					
	1	184 (45.8)		60.8 (54.5-67.1)	
	2-5	190 (47.3)		35.4 (29.1-41.6)	
	＞5	28 (7.0)		3.9 (1.9-5.9)	

^a^RDS: respondent-driven sampling.

^b^Weighted percentages.

### Descriptive and Correlation Analysis

The average scores of sexual compulsivity and sexual sensation seeking were 17.43 (SD 6.63) and 24.03 (SD 7.67), respectively. The average scores of multiple sex partners and unprotected anal intercourse were 2.51 (SD 3.61) and 4.29 (SD 1.13), respectively. The average score of gay app use was 3.17 (SD 2.02). Correlation analysis showed that sexual compulsivity was positively correlated with gay app use (*r*=0.11; *P*=.03) and multiple sex partners (*r*=0.25; *P*<.001). Sexual sensation seeking was positively correlated with gay app use (*r*=0.11; *P*=.31) and multiple sex partners (*r*=0.24; *P*<.001). Gay app use was positively correlated with both multiple sex partners (*r*=0.24; *P*<.001) and unprotected anal intercourse (*r*=0.15; *P*=.004), which were correlated with each other (*r*=0.24; *P*<.001). The detailed results are presented in Table 3.

**Table 3:** Descriptive statistics and bivariate relationships of key variables (N=402) of the cross-sectional study among men who have sex with men in China from August 1 to October 30, 2020.

Descriptive	Mean (SD)	1	2	3	4	5
Sexual compulsivity	17.43 (6.63)	1.00	N/A^a^	N/A	N/A	N/A
Sexual sensation seeking	24.03 (7.67)	0.64^b^	1.00	N/A	N/A	N/A
Gay app use	3.17 (2.02)	0.11^c^	0.11^c^	1.00	N/A	N/A
Multiple sex partners	2.51 (3.61)	0.25^b^	0.24^b^	0.24^b^	1.00	N/A
Unprotected anal intercourse	4.29 (1.13)	0.10	0.01	0.15^d^	0.02	1.00

^a^N/A: not applicable.

^b^*P*<.001.

^c^*P*<.05.

^d^*P*<.01.

### Mediation Effect Analysis

**Figure 3:**
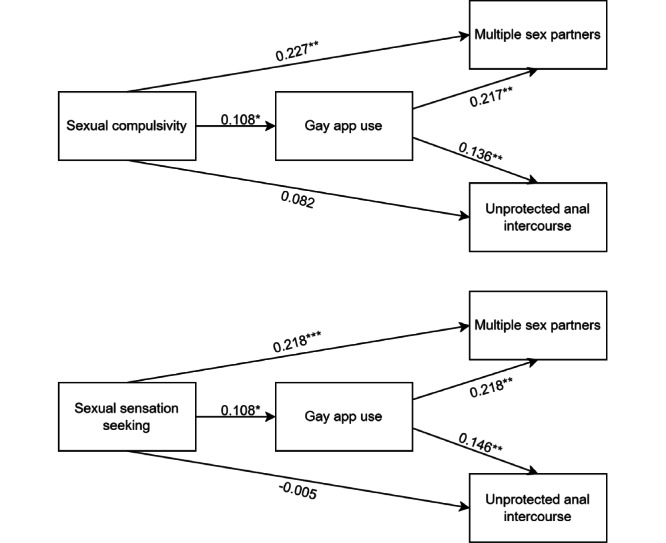
Visualization of path analysis results of the cross-sectional study among men who have sex with men in China from August 1 to October 30, 2020. *P<.05; **P<.01; and ***P<.001.

Figure 3 and Table 4 show the results of mediation analyses of gay app use on the relationship between sexuality traits (sexual compulsivity and sexual sensation seeking, respectively) and high-risk sexual behaviors (multiple sexual partners and unprotected anal intercourse, respectively). As shown in Table 4 and Figure 3, there was a significant direct (effect=0.227, 95% CI 0.070-0.358; *P*=.002) and indirect effect (effect=0.023, 95% CI 0.004-0.067; *P*=.01) between sexual compulsivity and multiple sexual partners. Gay app use acted as a partial mediator, accounting for 9.2% of the total effect. Additionally, there was a significant indirect effect (effect=0.015, 95% CI 0.004-0.031; *P*=.01) between sexual compulsivity and unprotected anal intercourse, although the direct effect (effect=0.082, 95% CI 0.011-0.150; *P*=.06) was not found to be significant. These results suggest that gay app use acts as a complete mediator in this relationship.

**Table 4:** Mediation analyses of gay app use between sexuality traits and high-risk sexual behaviors of the cross-sectional study among men who have sex with men in China from August 1 to October 30, 2020^a^.

Pathway	Total effect		Direct effect			Indirect effect					
	c^b^ (95% CI)	*P* value	c’^c^ (95% CI)	*P* value	a^d^ (95% CI)		*P* value	b^e^ (95% CI)	*P* value	a×b^f^ (95% CI)	*P* value
SC^g^→gay app use→ MSP	0.250 (0.103-0.367)	<.001	0.227 (0.070-0.358)	.002	0.108 (0.031-0.184)		.02	0.217 (0.094-0.410)	.002	0.023 (0.004-0.067)	.01
SC→gay app use→UAI^h^	0.097 (0.027-0.163)	.02	0.082 (–0.002 to 0.167)	.06	0.108 (0.031-0.184)		.02	0.136 (0.056-0.205)	.003	0.015 (0.004-0.031)	.01
SSS^i^→gay app use→MSP^j^	0.241 (0.126-0.326)	.002	0.218 (0.098-0.305)	<.001	0.108 (0.014-0.205)		.03	0.218 (0.102-0.409)	.002	0.023 (0.003-0.068)	.03
SSS→gay app use→UAI	0.011 (–0.080 to 0.107)	.81	–0.005 (–0.101 to 0.094)	.93	0.108 (0.014-0.205)		.03	0.146 (0.057-0.233)	.002	0.016 (0.001-0.038)	.03

^a^All models adjust for age, marital status, education, employment, monthly income, sexual orientation, and living status.

^b^Total effect.

^c^Direct effect (independent variables→dependent variables).

^d^Independent variables→mediator.

^e^Mediator→dependent variables.

^f^Indirect effect.

^g^SC: sexual compulsivity.

^h^UAI: unprotected anal intercourse.

^i^SSS: sexual sensation seeking.

^j^MSP: multiple sexual partners.

As shown in Table 4 and Figure 3, there was a significant direct effect (effect=0.218, 95% CI 0.098-0.305; *P*<.001) and indirect effect (effect=0.023, 95% CI 0.003-0.068; *P*=.03) between sexual sensation seeking and multiple sexual partners. Gay app use acted as a partial mediator, accounting for 9.5% of the total effect. Additionally, there was a significant indirect effect (effect=0.016, 95% CI 0.001-0.038; *P*=.03) between sexual sensation seeking and unprotected anal intercourse, but no significant direct effect (effect=–0.005, 95% CI –0.101 to 0.094; *P*=.93). These results suggest that gay app use plays a complete mediating role in this relationship. In addition, no stratified heterogeneity was found in the samples from both Wuhan and Changsha. Please refer to Multimedia Appendices 3 and 4 for details.

## Discussion

### Principal Findings

Drawing from the uses and gratifications theory, this study explored the mechanisms that underlie high-risk behaviors among MSM at the behavioral and psychosocial levels. The study’s findings indicated that sexuality traits (including sexual compulsivity and sexual sensation seeking) had a direct impact on high-risk behaviors (including multiple sexual partners and unprotected anal intercourse) among MSM and that the use of gay apps mediated the relationship between sexuality traits and high-risk behaviors.

A major finding was that sexuality traits had a direct influence on high-risk sexual behaviors. Specifically, MSM with higher levels of sexual compulsivity and sexual sensation-seeking were more likely to have multiple sexual partners. This finding was consistent with prior studies showing that sexual compulsivity and sexual sensation-seeking were positively associated with multiple sexual partners, unprotected anal intercourse, sexual acts under drug influence, and infections of HIV and other STIs [34-37]. Individuals with high sexual compulsivity cannot control their sexual thoughts and behaviors, they engage in frequent sexual activities and nonparaphilic activities, such as sex with anonymous partners, which all contribute to an increased risk of high-risk sexual behaviors [38,39]. Moreover, sexual sensation seekers are more likely to engage in sexual-related activities and behaviors that require increasing stimulation and adventure, leading to high-risk sexual behaviors [40]. Our findings suggest that sexuality traits are important risk factors for high-risk sexual behaviors among MSM and should be incorporated into HIV/AIDS prevention and intervention programs.

Another major finding was that gay app use mediated the relationship between sexuality traits and high-risk behaviors. Specifically, MSM with higher levels of sexual compulsivity and sexual sensation-seeking tend to spend more time and energy on gay apps, which in turn, lead to more high-risk sexual behaviors, such as having more sexual partners and engaging in more unprotected anal intercourse. This finding was in line with the uses and gratifications theory proposing that individuals follow their interests to choose media to satisfy their needs. According to the uses and gratifications theory, MSM’s gay app use (communication behavior) is determined by their sexual traits such as sexual compulsivity and sexual sensation-seeking (needs and motives). In order to satisfy their excessive sexual needs, they will engage in a series of high-risk behaviors such as multiple sexual partners and unprotected anal intercourse (the consequences of such behavior) [22]. Our findings suggest that the use of gay apps increases the likelihood of high-risk sexual behaviors among MSM with more prominent sexuality traits. Therefore, it is important to develop intervention programs that are based on gay apps for MSM. Moreover, interventions conducted through specially designed gay apps may also help reduce cultural and communication barriers between MSM and health care providers [41].

Our study showed that approximately 67% (n=271) of MSM used gay apps in the past 6 months to seek potential sexual partners in their local areas. This result confirms previous findings that MSM are increasingly using mobile internet to find sexual partners in the current era [42]. GPS-enabled social networking apps on smartphones allow users to find nearby sexual partners in a flexible and real-time manner and provide more opportunities for anonymous partner-seeking, which also increases the risk of high-risk behaviors among MSM [43,44]. However, other studies have demonstrated that gay apps may increase social support among this population, facilitate the provision of HIV testing services, and reduce related risky behaviors [45]. In recent years, with the advancement in artificial intelligence, some new apps have been developed to promote behavioral changes for HIV control among MSM. For example, Chen et al [46] proposed a chatbot for HIV self-testing to offer personalized, engaging, and needs-based health interventions to MSM based on human-machine interactions in Hongkong, which is expected to be equally, if not more, efficacious as HIV self-testing through live chat app. The use of gay apps can potentially have both favorable and unfavorable health effects, and it is imperative to emphasize the motivations and objectives of MSM using these apps, as they may result in distinct health outcomes.

This study reveals that in the past 6 months, 37% (n=149) of MSM have engaged in unprotected anal intercourse, and 54% (n=218) of MSM have had multiple sexual partners. These findings suggest a high prevalence of high-risk sexual behavior among MSM. The prevalence of unprotected anal intercourse among MSM in our study was higher than the reported 33% in a meta-analysis in China in 2014 [47], indicating an increasing trend of unprotected anal intercourse that warrants attention and action. A possible reason for this rise in prevalence may be related to the changes in the COVID-19 pandemic and related control measures over time. Studies have shown that high-risk sexual behaviors among MS decreased during the COVID-19 pandemic due to strict lockdown and social isolation measures, and then rebounded to the level before the COVID-19 outbreak after the pandemic received initial control in China [48]. The prevalence of multiple sexual partners in our study was consistent with that of previous studies. Unprotected anal intercourse and multiple sexual partners are identified as significant risk factors for HIV and other STIs among MSM [49,50]. It is important to continue monitoring these high-risk sexual behaviors in this population and take appropriate warning measures and contingency plans.

Wave-to-wave balance can be used to assess the balance of the recruited samples in the RDS process, which is crucial for estimating completion and evaluating the effectiveness of RDS [51]. The results showed that the primary indicators of this study eventually achieved balance over time. While the seeds chosen in the early stages of this study were not randomly selected, representative samples were recruited by selecting seeds from different cities and professions and ensuring that the social networks of seeds did not overlap, thereby achieving the goal of probability sampling.

There are several limitations to this study. First, the participants were recruited through the internet, and most of them were young and experienced with social media use. Therefore, this study may not represent MSM who are older or not familiar with social media, resulting in sample selection bias. The sample representativeness may be further limited by the RDS method used in this study, which may introduce statistical inference error due to the specific population, the way of sampling, and the method of estimation, referred to as the “statistical trinity” or the “spatial statistical trinity” [52]. However, RDS remains a valuable tool for researching hidden, hard-to-reach populations, and small subgroups that lack a well-defined sampling frame [53]. Second, all behavioral and psychosocial variables were self-reported, which could introduce social desirability bias. Finally, the study design was cross-sectional, making it difficult to establish causal relationships. In the future, more comprehensive and rigorous longitudinal studies should be conducted among MSM to determine the causal relationships between sexuality traits, gay app use, and the occurrence of high-risk behaviors.

### Conclusions

The study shows that high-risk sexual behaviors are common among MSM. Therefore, public health initiatives should prioritize prevention efforts that target these behaviors. Most MSM rely on gay apps to find sexual partners, which, when combined with higher levels of sexual compulsivity and sexual sensation seeking, can increase the likelihood of engaging in high-risk sexual behaviors. Therefore, interventions aimed at reducing these behaviors among MSM should focus on addressing the use of gay apps, while also considering the influence of their sexuality traits on gay app use.

## Appendix

Multimedia Appendix 1 Respondent-driven sampling (RDS) recruitment information.

Multimedia Appendix 2 The regional map of participants' household registrations by province.

Multimedia Appendix 3 Mediation analyses of gay app use between sexuality traits and high-risk sexual behaviors in Wuhan.

Multimedia Appendix 4 Mediation analyses of gay app use between sexuality traits and high-risk sexual behaviors in Changsha.

## Abbreviations

CDC: Center for Disease Control and Prevention
MSM: men who have sex with men
NGO: nongovernmental organization
RDS: respondent-driven sampling
SCS: Sexual Compulsivity Scale
SSSS: sexual sensation seeking scale
STI: sexually transmitted infection

## Data Availability

The data sets generated and analyzed during this study are not publicly available due to the sensitive nature of the MSM population in our study. In addition, the specific informed consent provided to participants also outlined that data would not be shared. However, data may be made available from the corresponding author upon reasonable request.

## References

[1] (2020). Annals of information on comprehensive prevention and treatment for AIDS, STD and hepatitis C. National Center for AIDS/STD Control and Prevention TCCfDCaPC.

[2] He HJC, Lyu P, Luan RS, Liao QH, Chang ZJ, Li Y, Ouyang L, Yang J (2016). Influence of sociocultural factors on HIV transmission among men who have sex with men: a qualitative study. Zhonghua Yu Fang Yi Xue Za Zhi.

[3] Dong MJ, Peng B, Liu ZF, Ye QN, Liu H, Lu XL, Zhang B, Chen JJ (2019). The prevalence of HIV among MSM in China: a large-scale systematic analysis. BMC Infect Dis.

[4] Liu Z, Chen Y, Yao T, Zhang T, Song D, Liu Y, Yu M, Xu J, Li Z, Yang J, Cui Z, Li C, Ma J (2021). Factors related to HIV testing frequency in MSM based on the 2011-2018 survey in Tianjin, China: a hint for risk reduction strategy. BMC Public Health.

[5] Zou H, Hu N, Xin Q, Beck J (2012). HIV testing among men who have sex with men in China: a systematic review and meta-analysis. AIDS Behav.

[6] Disman C (2003). The San Francisco bathhouse battles of 1984: civil liberties, AIDS risk, and shifts in health policy. J Homosex.

[7] Zheng JD, Pang L, Wu ZY (2008). The effect of internet on risk behavior among MSM and AIDS control and prevention. Chin J Health Educ.

[8] Yang G, Long J, Luo D, Xiao S, Kaminga AC (2019). The characteristics and quality of mobile phone apps targeted at men who have sex with men in China: a window of opportunity for health information dissemination?. JMIR Mhealth Uhealth.

[9] Wang S (2020). Chinese affective platform economies: dating, live streaming, and performative labor on blued. Media Cult Soc.

[10] Queiroz AAFLN, de Sousa ÁFL, de Araújo TME, de Oliveira FBM, Moura MEB, Reis RK (2017). A review of risk behaviors for HIV infection by men who have sex with men through geosocial networking phone apps. J Assoc Nurses AIDS Care.

[11] Holloway IW, Pulsipher CA, Gibbs J, Barman-Adhikari A, Rice E (2015). Network influences on the sexual risk behaviors of gay, bisexual and other men who have sex with men using geosocial networking applications. AIDS Behav.

[12] Grosskopf NA, LeVasseur MT, Glaser DB (2014). Use of the internet and mobile-based "apps" for sex-seeking among men who have sex with men in New York City. Am J Mens Health.

[13] Lehmiller JJ, Ioerger M (2014). Social networking smartphone applications and sexual health outcomes among men who have sex with men. PLoS One.

[14] Coleman E (1987). Sexual compulsivity: definition, etiology, and treatment considerations. J Chem Dep Treat.

[15] Muench F, Parsons JT (2004). Sexual compulsivity and HIV: identification and treatment. Focus.

[16] Derbyshire KL, Grant JE (2015). Compulsive sexual behavior: a review of the literature. J Behav Addict.

[17] Kalichman SC, Johnson JR, Adair V, Rompa D, Multhauf K, Kelly JA (1994). Sexual sensation seeking: scale development and predicting AIDS-risk behavior among homosexually active men. J Pers Assess.

[18] Matarelli SA (2013). Sexual sensation seeking and internet sex-seeking of middle eastern men who have sex with men. Arch Sex Behav.

[19] Kelly BC, Bimbi DS, Nanin JE, Izienicki H, Parsons JT (2009). Sexual compulsivity and sexual behaviors among gay and bisexual men and lesbian and bisexual women. J Sex Res.

[20] Shuper PA, Joharchi N, Rehm J (2014). Personality as a predictor of unprotected sexual behavior among people living with HIV/AIDS: a systematic review. AIDS Behav.

[21] Ruggiero TE (2000). Uses and gratifications theory in the 21st century. Mass Commun Soc.

[22] Rubin AM, Bryant J, Zillmann D (1994). Media uses and effects: a uses-and-gratifications perspective. Media Effects: Advances in Theory and Research.

[23] Hu M, Xu C, Wang J (2020). Spatiotemporal analysis of men who have sex with men in mainland China: social app capture-recapture method. JMIR Mhealth Uhealth.

[24] Zhao J, Cai R, Chen L, Cai W, Yang Z, Richardus JH, de Vlas SJ (2015). A comparison between respondent-driven sampling and time-location sampling among men who have sex with men in Shenzhen, China. Arch Sex Behav.

[25] Harris TG, Wu Y, Parmley LE, Musuka G, Mapingure MP, Chingombe I, Mugurungi O, Hakim A, Gozhora P, Miller SS, Lamb MR, Samba C, Rogers JH (2022). HIV care cascade and associated factors among men who have sex with men, transgender women, and genderqueer individuals in Zimbabwe: findings from a biobehavioural survey using respondent-driven sampling. Lancet HIV.

[26] McLaughlin KR, Johnston LG, Gamble LJ, Grigoryan T, Papoyan A, Grigoryan S (2019). Population size estimations among hidden populations using respondent-driven sampling surveys: case studies from Armenia. JMIR Public Health Surveill.

[27] Kendall C, Kerr LRFS, Gondim RC, Werneck GL, Macena RHM, Pontes MK, Johnston LG, Sabin K, McFarland W (2008). An empirical comparison of respondent-driven sampling, time location sampling, and snowball sampling for behavioral surveillance in men who have sex with men, Fortaleza, Brazil. AIDS Behav.

[28] Heckathorn DD (1997). Respondent-driven sampling: a new approach to the study of hidden populations. Soc Probl.

[29] Kalichman SC, Rompa D (1995). Sexual sensation seeking and sexual compulsivity scales: validity, and predicting HIV risk behavior. J Pers Assess.

[30] Parsons JT, Bimbi D, Halkitis PN (2001). Sexual compulsivity among gay/bisexual male escorts who advertise on the internet. Sex Addict Compulsivity.

[31] Ni Y, Liu H, Gong R, Shi M, Zhang S, Wang S, Cai Y (2021). The role of sexual compulsivity in unprotected intercourse among STI patients in Shanghai, China. BMC Public Health.

[32] Xu Y, Zheng Y, Rahman Q (2017). The relationship between self-reported sexually explicit media consumption and sexual risk behaviors among men who have sex with men in China. J Sex Med.

[33] Ramirez-Valles J, Heckathorn DD, Vázquez R, Diaz RM, Campbell RT (2005). The fit between theory and data in respondent-driven sampling: response to heimer. AIDS Behav.

[34] O'Leary A, Wolitski RJ, Remien RH, Woods WJ, Parsons JT, Moss S, Lyles CM (2005). Psychosocial correlates of transmission risk behavior among HIV-seropositive gay and bisexual men. AIDS.

[35] Grov C, Parsons JT, Bimbi DS (2010). Sexual compulsivity and sexual risk in gay and bisexual men. Arch Sex Behav.

[36] Dilley JW, Loeb L, Marson K, Chen S, Schwarcz S, Paul J, McFarland W (2008). Sexual compulsiveness and change in unprotected anal intercourse: unexpected results from a randomized controlled HIV counseling intervention study. J Acquir Immune Defic Syndr.

[37] Benotsch EG, Kalichman S, Cage M (2002). Men who have met sex partners via the internet: prevalence, predictors, and implications for HIV prevention. Arch Sex Behav.

[38] Walton MT, Bhullar N (2018). Hypersexuality, higher rates of intercourse, masturbation, sexual fantasy, and early sexual interest relate to higher sexual excitation/arousal. Arch Sex Behav.

[39] Slavin MN, Blycker GR, Potenza MN, Bőthe B, Demetrovics Z, Kraus SW (2020). Gender-related differences in associations between sexual abuse and hypersexuality. J Sex Med.

[40] Roberti JW (2004). A review of behavioral and biological correlates of sensation seeking. J Res Pers.

[41] Li C, Xiong Y, Sit HF, Tang W, Hall BJ, Muessig KE, Wei C, Bao H, Wei S, Zhang D, Mi G, Yu F, Tucker JD (2020). A men who have sex with men-friendly doctor finder hackathon in Guangzhou, China: development of a mobile health intervention to enhance health care utilization. JMIR Mhealth Uhealth.

[42] Goedel WC, Duncan DT (2015). Geosocial-networking app usage patterns of gay, bisexual, and other men who have sex with men: survey among users of grindr, a mobile dating app. JMIR Public Health Surveill.

[43] Li Q, Liu Y, Zhou Z, Li S, Luo F, Li D, Shi W, Jiang S, Yang Y, Jia Y, Xing H, Xiao D, Ruan Y, Shao Y (2012). Online sex-seeking behaviors among men who have sex with men: implications for investigation and intervention. AIDS Behav.

[44] Fan S, Li P, Hu Y, Gong H, Yu M, Ding Y, Luo Z, Wu G, Ouyang L, Zou H (2022). Geosocial networking smartphone app use and high-risk sexual behaviors among men who have sex with men attending university in China: cross-sectional study. JMIR Public Health Surveill.

[45] Klein H (2008). HIV risk practices sought by men who have sex with other men, and who use internet websites to identify potential sexual partners. Sex Health.

[46] Chen S, Zhang Q, Chan CK, Yu FY, Chidgey A, Fang Y, Mo PKH, Wang Z (2023). Evaluating an innovative HIV self-testing service with web-based, real-time counseling provided by an artificial intelligence chatbot (HIVST-chatbot) in increasing HIV self-testing use among Chinese men who have sex with men: protocol for a noninferiority randomized controlled trial. JMIR Res Protoc.

[47] Yang Z, Zhang S, Dong Z, Jin M, Han J (2014). Prevalence of unprotected anal intercourse in men who have sex with men recruited online versus offline: a meta-analysis. BMC Public Health.

[48] Zhang K, Chen S, Chan PSF, Fang Y, Cao H, Chen H, Hu T, Chen Y, Zhou X, Wang Z (2022). Changes in HIV testing utilization among Chinese men who have sex with men during the COVID-19 pandemic in Shenzhen, China: an observational prospective cohort study. Front Med (Lausanne).

[49] Beyrer C (2007). HIV epidemiology update and transmission factors: risks and risk contexts–16th international AIDS conference epidemiology plenary. Clin Infect Dis.

[50] Chu ZX, Xu JJ, Zhang YH, Zhang J, Hu QH, Yun K, Wang HY, Jiang YJ, Geng WQ, Shang H (2018). Poppers use and sexual partner concurrency increase the HIV incidence of MSM: a 24-month prospective cohort survey in Shenyang, China. Sci Rep.

[51] Heckathorn DD, Cameron CJ (2017). Network sampling: from snowball and multiplicity to respondent-driven sampling. Annu Rev Sociol.

[52] Wang J, Gao B, Stein A (2020). The spatial statistic trinity: a generic framework for spatial sampling and inference. Environ Model Softw.

[53] Raifman S, DeVost MA, Digitale JC, Chen YH, Morris MD (2022). Respondent-driven sampling: a sampling method for hard-to-reach populations and beyond. Curr Epidemiol Rep.

