# Supplementary material for: Gay App Use, Sexuality Traits, and High-Risk Sexual Behaviors Among Men Who Have Sex With Men in China: Mediation Analysis
Source: J Med Internet Res. 2023 Nov 1;25:e49137. doi: 10.2196/49137 (PMC10652192; doi:10.2196/49137)
Supplement: Multimedia Appendix 1 [file jmir_v25i1e49137_app1.docx]

Table S1. RDS recruitment information.

| **Variables** | **RDS crude** | | **RDS adjusted** | |
| --- | --- | --- | --- | --- |
|  | n | n/N (%) | n/N (%) | 95% CI |
| **Relationship with referrer** |  |  |  |  |
| Sexual partner beyond 6 months | 39 | 9.9% | 10.3% | 6.0%, 14.7% |
| Sexual partner in the past 6 months | 31 | 7.8% | 6.6% | 3.0%, 11.2% |
| Boyfriend | 50 | 12.7% | 16.2% | 9.5%, 23.0% |
| Regular friend | 197 | 50.0% | 34.5% | 29.7%, 45.5% |
| Online friend | 77 | 19.6% | 32.1% | 18.2%, 45.9% |
| **Social network size** |  |  |  |  |
| 0-24 | 199 | 51.5% | 90.9% | 88.3%, 93.3% |
| 25 or more | 195 | 48.5% | 9.1% | 6.7%, 11.7% |
