# Supplementary figures and images for: Gay App Use, Sexuality Traits, and High-Risk Sexual Behaviors Among Men Who Have Sex With Men in China: Mediation Analysis
Source: J Med Internet Res. 2023 Nov 1;25:e49137. doi: 10.2196/49137 (PMC10652192; doi:10.2196/49137)

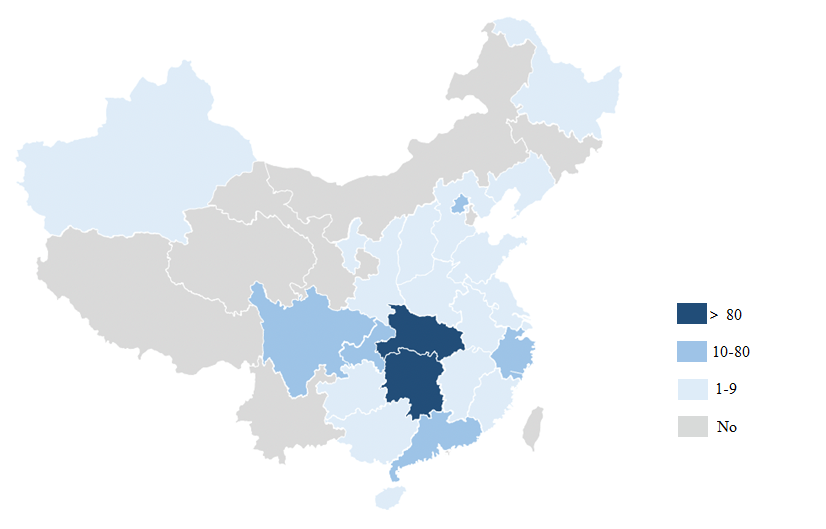

Supplement: Multimedia Appendix 2 [file jmir_v25i1e49137_app2.png]
