# Supplementary material for: Gay App Use, Sexuality Traits, and High-Risk Sexual Behaviors Among Men Who Have Sex With Men in China: Mediation Analysis
Source: J Med Internet Res. 2023 Nov 1;25:e49137. doi: 10.2196/49137 (PMC10652192; doi:10.2196/49137)
Supplement: Multimedia Appendix 3 [file jmir_v25i1e49137_app3.docx]

Table S3. Mediation analyses of gay app use between sexuality traits and high-risk sexual behaviors in Wuhan.

| Pathway | Total effect | | Direct effect |  | Indirect effect | | | | | |
| --- | --- | --- | --- | --- | --- | --- | --- | --- | --- | --- |
|  | c  (95%CI) | *P* | c’  (95%CI) | *P* | a  (95%CI) | *P* | b  (95%CI) | *P* | a*b  (95%CI) | *P* |
| SC →Gay app use→ MSP | 0.259  (0.041, 0.436) | .01 | 0.238  (0.005, 0.407) | .04 | 0.131  (0.004, 0.270) | .04 | 0.165  (0.038, 0.477) | .006 | 0.022  (0.001, 0.100) | .04 |
| SC →Gay app use→ UAI | 0.122  (0.006, 0.236) | .04 | 0.111  (-0.005, 0. 224) | .06 | 0.131  (0.004, 0.270) | .04 | 0.084  (0.057, 0.212) | .02 | 0.011  (0.004, 0.023) | .02 |
| SSS →Gay app use→ MSP | 0.239  (0.050, 0.381) | .002 | 0.219  (0.014, 0.385) | .03 | 0.119  (0.002, 0.262) | .04 | 0.170  (0.043, 0.474) | .002 | 0.020  (0.003, 0.046) | .01 |
| SSS →Gay app use→ UAI | 0.102  (-0.034, 0.232) | .23 | 0.091  (-0.042, 0.228) | .17 | 0.119  (0.002, 0.262) | .04 | 0.087  (0.051, 0.218) | .02 | 0.010  (0.003, 0.037) | .02 |
