# Supplementary material for: Gay App Use, Sexuality Traits, and High-Risk Sexual Behaviors Among Men Who Have Sex With Men in China: Mediation Analysis
Source: J Med Internet Res. 2023 Nov 1;25:e49137. doi: 10.2196/49137 (PMC10652192; doi:10.2196/49137)
Supplement: Multimedia Appendix 4 [file jmir_v25i1e49137_app4.docx]

Table S2. Mediation analyses of gay app use between sexuality traits and high-risk sexual behaviors in Changsha.

| Pathway | Total effect | | Direct effect |  | Indirect effect | | | | | |
| --- | --- | --- | --- | --- | --- | --- | --- | --- | --- | --- |
|  | c  (95%CI) | *P* | c’  (95%CI) | *P* | a  (95%CI) | *P* | b  (95%CI) | *P* | a*b  (95%CI) | *P* |
| SC →Gay app use→ MSP | 0.246  (0.084, 0.449) | <.001 | 0.218  (0.055, 0.428) | .004 | 0.086  (0.035, 0.141) | .02 | 0.330  (0.213, 0.437) | <.001 | 0.028  (0.010, 0.048) | .01 |
| SC →Gay app use→ UAI | 0.073  (0.001, 0.145) | .03 | 0.057  (-0.172, 0.077) | .417 | 0.086  (0.035, 0.141) | .02 | 0.185  (0.056, 0.290) | .003 | 0.016  (0.008, 0.034) | .02 |
| SSS →Gay app use→ MSP | 0.280  (0.122, 0.419) | .002 | 0.249  (0.097, 0.388) | <.001 | 0.094  (0.033, 0.155) | .01 | 0.249  (0.097, 0.388) | <.001 | 0.031  (0.012, 0.060) | .02 |
| SSS →Gay app use→ UAI | 0.085  (0.045,0.214) | .001 | 0.066  (-0.017, 0.212) | .112 | 0.094  (-0.041, 0.225) | .01 | 0.200  (0.077, 0.306) | .002 | 0.019  (0.007, 0.035) | .01 |

SC: Sexual compulsivity; SSS: Sexual sensation seeking; UAI: Unprotected anal intercourse; MSP: Multiple sexual partners.

All models adjust for age, marital status, education, employment, monthly income, sexual orientation, and living status.
